# Aging Response and Precipitation Behavior after 5% Pre-Deformation of an Al-Mg-Si-Cu Alloy

**DOI:** 10.3390/ma11081422

**Published:** 2018-08-13

**Authors:** Shuoxun Jin, Tungwai Ngai, Liejun Li, Shian Jia, Tongguang Zhai, Dongjie Ke

**Affiliations:** 1 Guangdong Key Laboratory for Processing and Forming of Advanced Metallic Materials, South China University of Technology, Guangzhou 510640, China; shuoxunjimmy@outlook.com (S.J.); dhni@scut.edu.cn (T.N.); 2 Department of Chemical and Materials Engineering, University of Kentucky, Lexington, KY 40506, USA; tongguang.zhai@uky.edu; 3 Center for Aluminum Technology, University of Kentucky, Lexington, KY 40511, USA; shian.jia@gmail.com; 4 Fujian Metal-New Aluminum Technology Co. Ltd. 3# Alloy Road, Wuhushan Industry Area Qingkou Investment Zone, Fuzhou 350000, China; kdj@vip.163.com

**Keywords:** Al-Mg-Si-Cu alloy, precipitation behavior, pre-deformation, mechanical properties

## Abstract

In this study, Al-1.00 Mg-0.65 Si-0.24 Cu alloy was solution heat-treated, water-quenched, and then pre-deformed for 5% before aging. The peak hardness and yield strength of the pre-deformed sample with subsequent artificial aging were similar to that of a T6 condition sample. It was also found that the pre-deformation treatment could inhibit the negative influence of natural aging to some degree. After seven days of natural aging, the pre-deformed sample obtained better peak hardness and yield strength upon artificial aging than the sample without pre-deformation. In addition, the pre-deformation treatment could reduce 50% of the artificial aging time to reach the peak aging condition compared with T6 treatment. For the peak aged condition in the pre-deformed sample, transmission electron microscopy (TEM) observation found two types of precipitates exhibited along the dislocations besides the β″ precipitates in the Al matrix. Both precipitates had disordered atomic arrangements on the ordered subcell (Si network). The disordered precipitates occupied a number of Mg and Si atoms, resulting in less β″ precipitates formed during artificial aging at 180 °C.

## 1. Introduction

The 6xxx series Al-Mg-Si-Cu alloy is an important series of Al alloys for application as structural materials [1,2]. Precipitation hardening is one of the key mechanisms that enable Al-Mg-Si-Cu alloys to provide better properties [3,4].

Edwards et al. [5] reported the evolution of metastable phases in the Al-Mg-Si (-Cu) alloys as: supersaturated solid solution → Mg/Si clusters → β″ → β′ → β. Fallah et al. [6] revealed that Si-rich and Mg-rich clusters started to form in several hours after natural aging. Then, the fine needle-shaped β″ phase started to appear at around 180 °C [7,8]. It has been proven that the β″ phase is very effective in strengthening the Al-Mg-Si alloy matrix [9]. Vissers et al. [10] studied the crystal structure of the β′ phase, and reported that the β′ phase has a hexagonal unit cell, in which a = 0.715 nm and c = 1.215 nm. Recent studies have investigated the effect of Cu on the precipitation hardening of Al-Mg-Si-Cu alloys. Xiao et al. [11] investigated the precipitation hardening process with different Cu composition in Al-Mg-Si-Cu alloys, revealing that more Cu content results in a more significant age hardening response, and the Q′ phase was formed when the Cu content was larger than 1.0 wt %. Ding et al. [12] found that the alloy with the composition of Al-1.17Mg-0.55Si-0.47Cu has better mechanical properties, making it more suitable for auto body panel applications.

After solution heat treatment, the aluminum materials are usually stored for more than one week at room temperature before being put into further application, during which aluminum undergoes a natural aging process (NA) [13]. Pogatscher et al. [14,15] found that NA impacts the properties of Al-Mg-Si alloys in different ways, depending on the Mg and Si content in the alloy. It has been reported that NA performed a positive effect on the subsequent artificial aging process (AA) when the total solute content of Mg and Si is lower than 1 wt % [16]. Aruga et al. [17] reported that NA exerts a significant negative influence on the subsequent artificial aging process when the solute content (Mg + Si) is beyond 1 wt % in Al-Mg-Si (-Cu) alloys. After quenching, Mg and Si atoms start to precipitate immediately during NA. The clusters that formed during NA would stop elongating at an early stage, resulting in a size too small to act as nucleation sites for strengthen phases [18]. Moreover, Marioara et al. [19] found that the clusters that formed at an early stage of NA process can neither dissolve into aluminum matrix nor grow larger during subsequent AA, negatively affecting aging hardening and thus mechanical properties such as peak hardness in a 6082 aluminum alloy. Jin et al. [7] showed that NA could reduce the size and volume fraction of β″ precipitates at a subsequent peak AA condition, which results in a significant decrease of peak hardness and a yield strength in 6061 aluminum alloy.

Some studies suggested that adopting deformation after water quenching could affect the precipitation for Al-Mg-Si-Cu alloy. The dislocations that are generated by deformation decreased the activation energy for precipitates and accelerated precipitate reaction [20]. Moreover, the deformation changed the precipitation of the β′ + Q′ phase instead of the β″ phase in AA6022 alloy [21]. Teichmann et al. [22] investigated the influence of pre-deformation on early-stage precipitation by high-resolution transmission electron microscopy (HRTEM) in 6060 aluminum alloy, and the results revealed that two types of disordered precipitates were grown on dislocation lines instead of β″ precipitates. However, it has been suggested that the deformed and undeformed samples have the same phase transformation sequence in an Al-Mg-Si-Cu alloy: solid solution α → Mg/Si clusters → β″ → β′ + Q′ → Q [23].

Up until now, the precipitation of Al-Mg-Si-Cu alloy after deformation is still the subject of many disputes. In addition, few studies have investigated the effect of deformation on the mechanical properties during subsequent AA, and it is also necessary to identify whether the negative influence of NA can be inhibited by introduced pre-deformation. In the present work, hardness measurement, tensile test, transmission electron microscopy (TEM), HRTEM, and scanning electron microscopy (SEM) analysis were adopted to study the effect of 5% pre-deformation on the mechanical properties and precipitation behavior upon the AA of an Al-Mg-Si-Cu alloy. The correlation between mechanical properties and microstructure was demonstrated, and the influence of pre-deformation on precipitation behavior has also been discussed.

## 2. Materials and Methods 

The material used in this work is standard AA6061 alloy, which is widely applied in commercial industry with the composition of 1.00 Mg-0.65 Si-0.24 Cu-0.14 Fe-balance Al (wt %). The cast ingots were homogenized, and then went through hot and cold rolling to achieve a 4.0-mm final gauge. The cold rolled sheets were solution heat-treated at 540 °C for 1 h, and then water quenched to room temperature, followed by cold rolling by 5% (pre-deformation treatment) within 10 min. After pre-deformation, the AA process was performed at 180 °C for 0–300 min, either with or without prior natural aging (NA). In the present work, the processes of the samples are listed in Table 1. 

The Vickers microhardness of the samples was performed with a 100-g load, and the holding time is 15 s. At least seven indentations were measured to get an average value of hardness. The tensile tests were carried out on the MTS 810 tensile testing machine (MTS Systems Corporation, Eden Prairie, MN, USA). The samples for tensile testing had a gauge length of 36 mm, a width of 10 mm, and a thickness of 4 mm. Three tensile samples were tested for each experimental state. 

The SEM studies were measured by a HITACHI s-4300 microscope (HITACHI Ltd., Tokyo, Japan) at 20 kV. The TEM specimens were firstly prepared by mechanical polishing and followed by electropolishing. The electrolyte consisted of 33% HNO_3_ in methanol and was kept at temperatures between −25 °C and −20 °C during thinning. The TEM and HRTEM investigations were performed on an FEI Tecnai F20 TEM (FEI Company, Hillsboro, OR, USA) operated at 200 kV. 

## 3. Results

### 3.1. Age-Hardening Behavior

**Figure 1:**
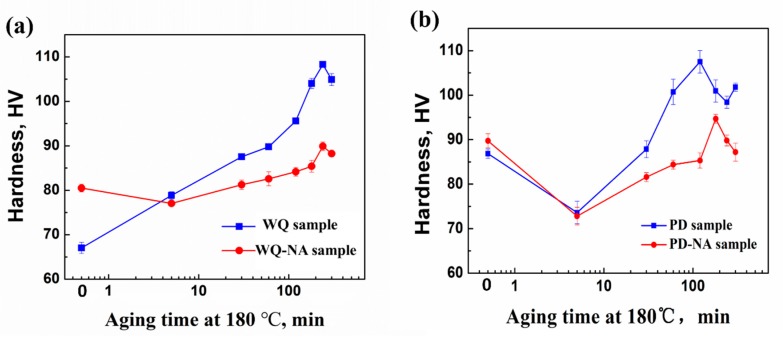
Age-hardening curves during artificial aging at 180 °C, either with or without prior natural aging: (**a**) samples were water quenched after solution; (**b**) samples with pre-deformation after quenching.

The changes in hardness during artificial aging at 180 °C for the samples are displayed in Figure 1. Figure 1a shows that the natural aging has a significant negative effect on the age-hardening response in the samples without pre-deformation. The WQ-NA sample is 13 HV higher than the WQ sample in hardness before artificial aging, which is a result of the clusters formed in the NA process. The WQ-NA sample has a 4-HV drop in hardness at the early stage of artificial aging, indicating that a part of the clusters formed in NA had dissolved upon AA. Through 30 min of artificial aging, the hardness in the WQ-NA sample becomes similar to the original hardness before artificial aging. Compared with the WQ-NA sample, the hardness of the WQ sample starts to increase over aging time, and reached 90 HV after 30 min AA. In the case of the longer AA time, both the WQ and WQ-NA samples reach the peak hardness around 240 min; however, the peak hardness of the WQ-NA sample is 21 HV lower than that of the WQ sample.

It can be seen in Figure 1b that the hardness of the samples increased after pre-deformation, indicating that the dislocations introduced by pre-deformation have an impact on hardness. Moreover, the hardness of the PD-NA sample is only 2 HV higher than that of the PD sample, which suggests that natural aging has a much lower influence on these samples compared to the samples without pre-deformation. In the first stage of AA, the hardness in the PD and PD-NA samples are both decreased; then, the hardness increased rapidly to reach peak hardness over the aging time. The peak hardness of the PD sample is similar with that of the WQ sample, and the the PD-NA sample is 5 HV higher than the WQ-HV sample in peak hardness. It is worth pointing out that the PD sample reaches peak hardness at 120 min AA, which is a 50% reduction in aging time compared to that of the WQ sample.

### 3.2. Tensile Behavior

**Figure 2:**
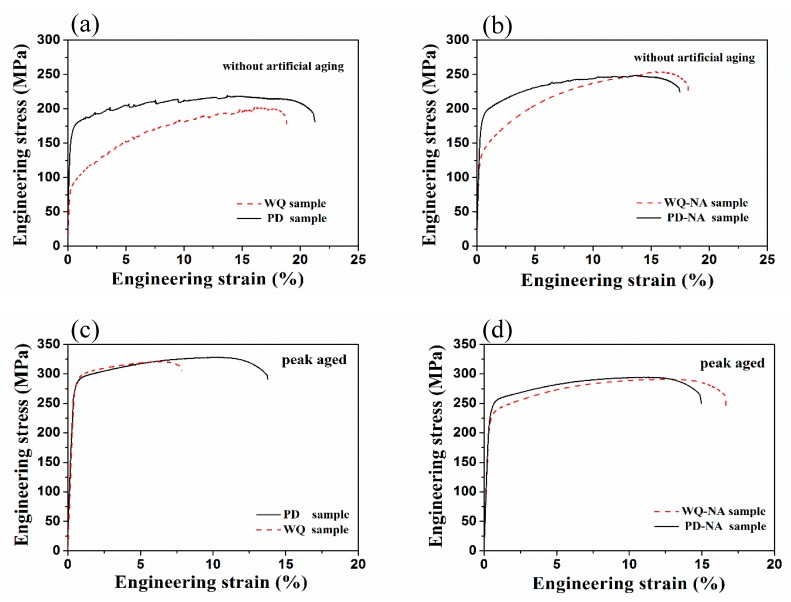
Engineering stress–engineering strain curves of the samples: (**a**) PD and WQ samples without artificial aging; (**b**) WQ-NA and PD-NA samples without artificial aging; (**c**) peak artificial aged PD and WQ samples; (**d**) peak artificial aged WQ-NA and PD-NA samples. NA: natural aging.

Figure 2 shows the engineering stress–engineering strain curves, and Table 2 shows the tensile data for samples treated by different processes. Figure 2a implies the curves of the WQ and PD samples without AA. To avoid the influence of natural aging, tensile tests were conducted immediately after quenching or pre-deformation (within five min). “Serrated flow” was observed on stress–strain curves. After seven days of NA process, the yield strength increased from 166.8 MPa to 183.4 MPa for the PD sample, and 84.8 MPa to 131.0 MPa for the WQ sample. As shown in Figure 2b, the serrated flow still can be detected in the curve of the PD-NA sample, and there is no sign of it in the curve of the WQ-NA sample. Reed et al. [24] provided an explanation on the “serrated flow” that it is commonly related to the interaction of moving dislocations and diffusing solute atoms. Considering that the serrations are the indication of the atoms being dissolved in the matrix, the pre-deformation treatment can inhibit the formation of NA clusters to a certain degree. These results suggest that NA has a strong influence on yield strength in the WQ sample, while the effect on the PD sample is much smaller.

Figure 2c,d exhibits the engineering stress-engineering strain curves of peak artificial aged samples. The yield strengths are similar in the peak artificial aged samples which are without prior natural aging (Figure 2c). For samples that were naturally aged for seven days, the yield strength of the PD-NA sample is 16.5 MPa higher than the WQ-NA sample after subsequent peak AA (Figure 2d). The elongations of the samples have all declined after peak AA; especially, the elongation of the WQ sample dropped from 19.8% to 7.8%. However, the elongation of the PD sample dropped much less than the WQ sample after peak AA. 

### 3.3. Fracture Mechanism

**Figure 3:**
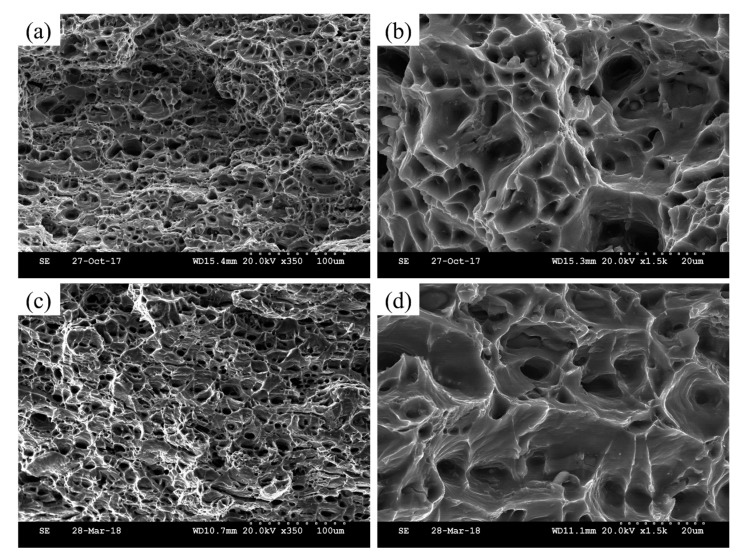
SEM fractographs of peak artificial aged samples: (**a**,**b**) WQ sample; (**c**,**d**) PD sample.

**Figure 4:**
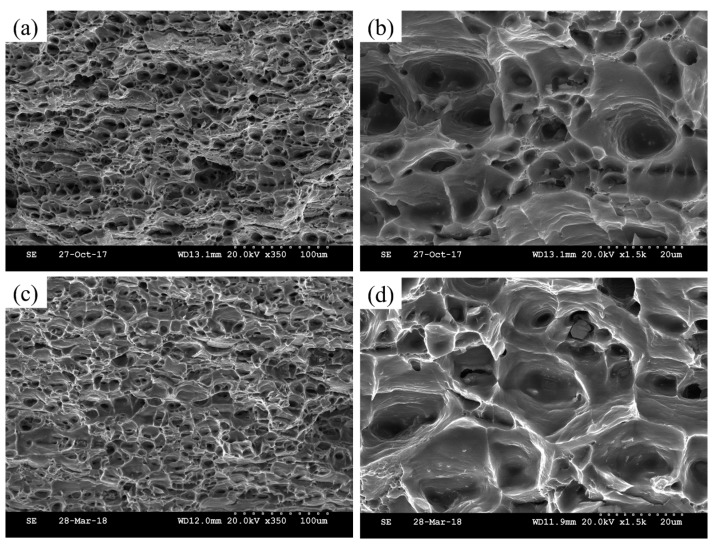
SEM fractographs of peak artificial aged samples: (**a**,**b**) WQ-NA sample; (**c**,**d**) PD-NA sample.

Figure 3 and Figure 4 show the SEM micrograph of tensile fracture in the peak-aged samples. As can be seen from the morphology of the WQ and PD samples (Figure 3), the fracture surface distributed a mount of dimples with different sizes, and no sign of river pattern was observed. The result implies that both samples have a typical ductile fracture. By comparison, the dimples in the PD sample are much deeper than that of the WQ sample. Figure 4 shows the fracture surface in the WQ-NA and PD-NA samples, and the same type of ductile fracture was observed. Both samples showed similar fracture structure, and the PD-NA sample showed slightly deeper dimples than the WQ-NA sample (Figure 3b). 

### 3.4. Precipitation Behavior

**Figure 5:**
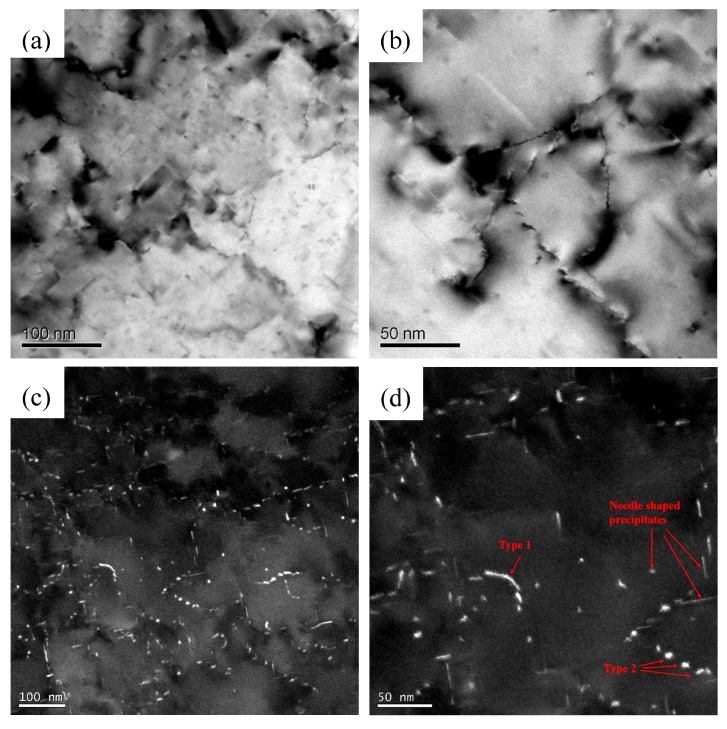
Precipitate microstructures of the PD sample after peak artificial aging at 180 °C: (**a**,**b**) TEM bright field images; (**c**,**d**) TEM dark field images.

To study the precipitation behavior of peak artificial aging condition (AA 120 min after pre-deformation) in the PD sample, TEM analyses were selected. All of the micrographs in this study were obtained at <001>_Al_ zone axes. Figure 5a,b illustrates that the TEM bright field of the PD sample artificially aged for 120 min at 180 ℃. Needle-shaped precipitates can be seen clearly in the matrix (Figure 5a). Previous work has studied the morphology of this precipitate carefully [7], indicating that the precipitates belonged to the β″ phase. The needle-shaped β″ phase is the most effective precipitate in the strengthening of the undeformed Al-Mg-Si-Cu alloy [9]. In addition, dislocations generated by the pre-deformation are present in the microstructure. Two types of precipitates exhibited along the dislocations can be seen in the enlarged bright field TEM image (Figure 5b). From the dark field TEM image (Figure 5c) and enlarged image (Figure 5d), the two types of precipitates along the dislocations can be identified. Type 1 is a series of precipitates discretely nucleated on the dislocations, and Type 2 are narrow, long, and curved precipitates along the dislocations.

**Figure 6:**
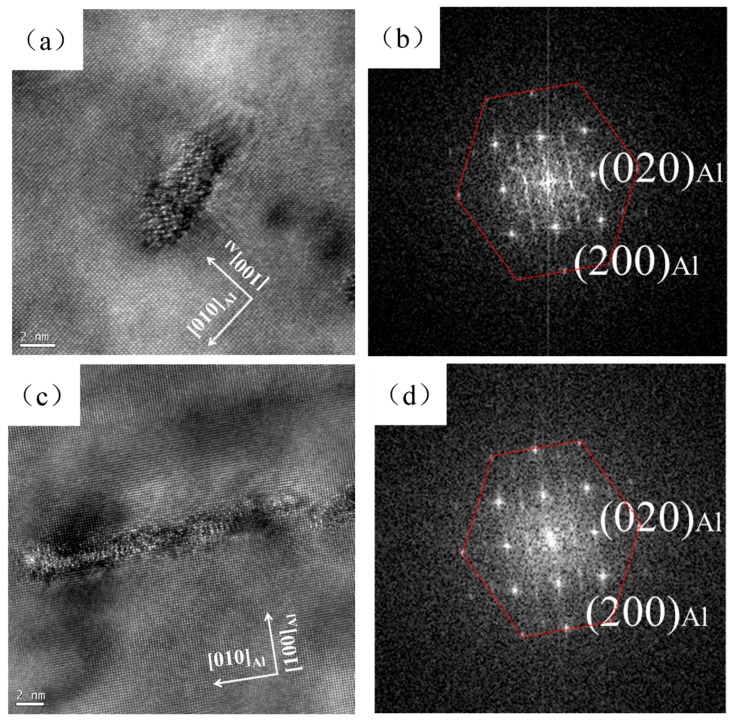
(**a**) High-resolution transmission electron microscopy (HRTEM) image of the Type 1 precipitate; (**b**) the corresponding fast Fourier transform (FFT) patterns of (**a**); (**c**) HRTEM image of the Type 2 precipitate; (**d**) the corresponding FFT patterns of (**c**).

Moreover, HRTEM analysis was carried out to investigate the Type 1 and Type 2 precipitates. As shown in Figure 6a,c, no ordered atomic arrangement was found in both Type 1 and Type 2 precipitates. The results of the corresponding fast Fourier transform (FFT) are also noisy except for the Al matrix (Figure 6b,d), indicating that the Type 1 and Type 2 precipitates are all disordered precipitates.

## 4. Discussion

The hardness has exhibited a marked increase after seven days of natural aging in the WQ sample (shown in Figure 1a). According to the three-dimensional (3D) atom probe investigations by Cao et al. [25], Mg and Si clusters could form rapidly within several hours during the NA process, which could explain the improvement in the hardness. Fallah et al. [6] further proposed that Si-rich clusters started to form in the matrix at the beginning of the NA process, followed by a part of these clusters attracting a large amount of Mg atoms and becoming Mg-rich clusters. All of the clusters had a FCC structure and were very small in size. Fallah et al. [4] continued to report that the Mg-rich clusters would dissolve into the Al matrix when the alloy reached 70 °C during the aging process. Since the Mg-rich clusters contributed to strengthening the alloy, so the dissolution of the Mg-rich clusters related to the marked hardness decrease at the beginning stage of the AA process (Figure 1a) of the WQ-NA sample. In addition, the peak hardness of the WQ-NA sample was much lower than that of the WQ sample. The previous study [7] reported that the β″ precipitates in the WQ-NA sample were significantly smaller in size and lower in volume fraction than those in the WQ sample at peak artificial aged condition, which results in the marked decline of peak hardness. 

For the pre-deformed samples, the hardness of the sample increased after 5% cold rolling (Figure 1b). However, the hardness appears to have only a slight increase (2 HV) after seven days of natural aging in the PD sample. Moreover, a small-scaled serrated flow still can be detected in the engineering stress–engineering strain curve of the PD-NA sample (Figure 2b), which is not founded in the curve of the WQ-NA sample. These results are believed that the pre-deformation treatment would suppress the formation of NA clusters, since the serrated flow is evidence that the Mg and Si atoms are dissolved in the Al matrix. The hardness curve (Figure 1b) and the engineering stress–engineering strain curve (Figure 2c) suggest that the hardness and yield strength in the peak artificial aged PD sample are similar to that of the peak artificial aged WQ sample. Meanwhile, the peak hardness and yield strength in the PD-NA sample are obviously higher than that of the WQ-NA sample (Figure 2d), indicating that the pre-deformation treatment could inhibit the negative effect of NA to a certain degree. Figure 2c,d shows that the elongation of the WQ sample drops more sharply than the other three states of samples after subsequent peak artificial aging. The SEM image of tensile fracture (Figure 3) shows that the dimples in the PD sample are much deeper than that of the WQ sample at peak aged condition. The dimples in the WQ-NA sample and PD-NA sample are similar to that of the PD sample at peak aged condition (Figure 4). The SEM results agree well with the differences in elongation; previous work [7] adopted TEM and HRTEM to find out that the β″ precipitates in the WQ-NA sample are significantly lower in volume fraction and smaller in size than that in the WQ sample at peak aged condition, which results in the deeper dimples and better elongation. The TEM study in this work (Figure 5) shows that another two types of precipitates exhibited along the dislocations besides the β″ precipitates in the Al matrix. The HRTEM images reveal that both the Type 1 and Type 2 precipitates are disordered precipitates. However, the hexagonal subsymmetry of the precipitate structures are clearly found in the FFT patterns (Figure 6b,d), which is similar to the Si network in the Al-Mg-Si (-Cu) alloys [22,26]. It is believed that both the Type 1 and Type 2 precipitates contain Si atomic columns. Liu et al. [27] found similar precipitates in a deformed Al-Mg-Si-Cu alloy, and proposed that the Mg/Si/Cu ratio of the disordered precipitates is about 4.5/3.4/1. Previous work [28] found the same precipitates, and reported that the precipitates that were generated from pretreatment contribute to the strengthening of the alloy. Fewer β″ precipitates were formed in the Al matrix during artificial aging, since many Mg and Si atoms are occupied by Type 1 and Type 2 precipitates; therefore, the PD and PD-NA samples display a better elongation than the WQ sample at peak aged condition. 

It is worth pointing out that the artificial aging time to reach peak condition is decreased by 50% in the PD sample. Quainoo and Yannacopoulos [29] suggested that the dislocations generated by pre-deformation are favorable heterogeneous nucleation sites for precipitates and a short path for diffusion, which tends to accelerate the process of precipitation. Yassar et al. [20] performed differential scanning calorimetric (DSC) analyses and adopted the Arrhenius-type equation to measure the activation energy for metastable phases:

(1) $$\frac{dY}{dt}=f\left(Y\right)k_{0}\exp\left(\frac{-Q*}{RT}\right)$$

where $\frac{dY}{dt}$ is the precipitation rate, *k*_0_ is the frequency factor, Q* is the activation energy, *R* is the gas constant, *T* is the absolute temperature, and *f*(*Y*) is only dependent on *Y*. The results suggest that deformation could significantly decrease the activation energy for precipitates. In summary, the dislocations introduced by pre-deformation are helpful for the formation of precipitates. On the other hand, the amount of solute atoms that are occupied by Type 1 and Type 2 precipitates result in less β′′ precipitates being formed at peak aged condition. Therefore, the pre-deformation treatment could reduce 50% of the AA time to reach the peak aging condition. 

## 5. Conclusions

In this work, SEM, TEM, HRTEM, hardness, and tensile measurements have been applied to investigate the effect of 5% pre-deformation on the mechanical properties and precipitation behavior upon AA of an Al-Mg-Si-Cu alloy. The following conclusions are obtained:

1. After quenching, 5% pre-deformation cannot increase the peak hardness and yield strength upon subsequent artificial aging compared with the T6 condition. However, the pre-deformation treatment could inhibit the negative influence of natural aging to some degree. After seven days of a natural aging process, the pre-deformed sample has better peak hardness and yield strength upon artificial aging.
2. The sample with 5% pre-deformation treatment reaches the peak artificial aging condition after 120 min, compared to 240 min of the sample without pre-deformation.
3. The pre-deformation treatment could reduce 50% of the artificial aging time to reach the peak aging condition compared with T6 treatment. The dislocations generated by pre-deformation significantly accelerate the process of precipitation during artificial aging.
4. Type 1 and Type 2 precipitates are exhibited along dislocations after pre-deformation, which have disordered atomic arrangements on the ordered subcell (Si network). The disordered precipitates occupied a number of Mg and Si atoms, and result in fewer β" precipitates being formed during subsequent artificial aging.

## Figures and Tables

**Table 1:** The processes of the samples.

Sample	Process of the Sample
WQ	Sample solution heat treatment at 540 °C for 1 h, and then water quenched to room temperature
WQ-NA	WQ sample then had seven days of natural aging
PD	WQ sample immediate cold rolling by 5% (pre-deformation)
PD-NA	PD sample then had seven days of natural aging

**Table 2:** Mechanical properties of the samples.

Sample	Yield Strength (MPa)	Ultimate Tensile Strength (MPa)	Elongation (%)
WQ	84.8 ± 1.8	202.0 ± 2.1	19.8 ± 2
PD	166.8 ± 3.8	219.9 ± 4.6	22.2 ± 1.2
WQ-NA	131.0 ± 5.1	254.4 ± 7.5	20.8 ± 1.3
PD-NA	183.4 ± 6.1	248.9 ± 3.2	17.2 ± 1.5
Peak aged WQ	287.5 ± 5.2	301.3 ± 7.2	7.8 ± 1.2
Peak aged PD	287.9 ± 4.3	318.2 ± 5.1	13.6 ± 2.1
Peak aged WQ-NA	229.6 ± 4.8	221.6 ± 5.6	16.5 ± 2.0
Peak aged PD-NA	246.1 ± 5.6	230.4 ± 6.2	15.4 ± 1.8
